# Multi-Scale Glycemic Variability: A Link to Gray Matter Atrophy and Cognitive Decline in Type 2 Diabetes

**DOI:** 10.1371/journal.pone.0086284

**Published:** 2014-01-24

**Authors:** Xingran Cui, Amir Abduljalil, Brad D. Manor, Chung-Kang Peng, Vera Novak

**Affiliations:** 1 Division of Interdisciplinary Medicine and Biotechnology, Beth Israel Deaconess Medical Center, Harvard Medical School, Boston, Massachusetts, United States of America; 2 Wright Center of Innovation, Dept. of Radiology, The Ohio State University, Columbus Ohio, United States of America; 3 Institute for Aging Research, Hebrew SeniorLife, Roslindale, Massachusetts, United States of America; 4 Division of Gerontology, Beth Israel Deaconess Medical Center, Harvard Medical School, Boston, Massachusetts, United States of America; 5 Center for Dynamical Biomarkers and Translational Medicine, National Central University, Chung-Li, Taiwan; 6 Division of Stroke, Dept. of Neurology, Beth Israel Deaconess Medical Center, Harvard Medical School, Boston, Massachusetts, United States of America; Beijing Normal University, China

## Abstract

**Objective:**

Type 2 diabetes mellitus (DM) accelerates brain aging and cognitive decline. Complex interactions between hyperglycemia, glycemic variability and brain aging remain unresolved. This study investigated the relationship between glycemic variability at multiple time scales, brain volumes and cognition in type 2 DM.

**Research Design and Methods:**

Forty-three older adults with and 26 without type 2 DM completed 72-hour continuous glucose monitoring, cognitive tests and anatomical MRI. We described a new analysis of continuous glucose monitoring, termed Multi-Scale glycemic variability (Multi-Scale GV), to examine glycemic variability at multiple time scales. Specifically, Ensemble Empirical Mode Decomposition was used to identify five unique ultradian glycemic variability cycles (GVC_1–5_) that modulate serum glucose with periods ranging from 0.5–12 hrs.

**Results:**

Type 2 DM subjects demonstrated greater variability in GVC_3–5_ (period 2.0–12 hrs) than controls (*P*<0.0001), during the day as well as during the night. Multi-Scale GV was related to conventional markers of glycemic variability (e.g. standard deviation and mean glycemic excursions), but demonstrated greater sensitivity and specificity to conventional markers, and was associated with worse long-term glycemic control (e.g. fasting glucose and HbA1c). Across all subjects, those with greater glycemic variability within higher frequency cycles (GVC_1–3_; 0.5–2.0 hrs) had less gray matter within the limbic system and temporo-parietal lobes (e.g. cingulum, insular, hippocampus), and exhibited worse cognitive performance. Specifically within those with type 2 DM, greater glycemic variability in GVC_2–3_ was associated with worse learning and memory scores. Greater variability in GVC_5_ was associated with longer DM duration and more depression. These relationships were independent of HbA1c and hypoglycemic episodes.

**Conclusions:**

Type 2 DM is associated with dysregulation of glycemic variability over multiple scales of time. These time-scale-dependent glycemic fluctuations might contribute to brain atrophy and cognitive outcomes within this vulnerable population.

## Introduction

Type 2 diabetes mellitus (type 2 DM) is among the leading causes of morbidity, cognitive decline and dementia [1], [2]. DM accelerates signs of brain aging and manifests as regional hypoperfusion, tissue atrophy and cognitive and functional impairment [3], [4]. Fluctuation of serum glucose between hyper- and hypoglycemic levels may exacerbate the risk for dementia [2], [5], [6]. Recent clinical trials indicate that interactions between chronic hyperglycemia and glycemic variability are complex and that their effects on the brain and cardiovascular autonomic system are not well understood [7], [8].

Glucose metabolism is influenced by numerous intrinsic rhythms and behaviors, including autonomic, hormonal and cardiovascular responses to activity, meals and sleep [9]–[12]. These regulatory processes often interact and are closely coupled with the body clock system and central autonomic neural networks, resulting in complex dynamics of glucose and insulin signaling between the brain and peripheral organs. Glucose variability is linked with several ultradian rhythms, e.g., insulin and cortisol secretion [13]–[17], autonomic control of sleep cycle and nighttime blood pressure reduction (i.e., nocturnal dipping) [12], [18], meals and sleep/wake cycles [12], [19]. Therefore, serum glucose levels fluctuate over multiple temporal scales, and alterations of these regulatory processes may contribute to type 2 DM-related complications [20], [21]. Recent advances in signal processing allowed to quantify multi-scale fluctuations from a discrete, non-stationary time-series of serum glucose and therefore may provide critical insight into the effects of glucose variability on functional outcomes.

Our objective was to study the effects of glycemic variability at distinct time scales on the brain and cognition in older diabetic adults.

We hypothesized that: 1) Type 2 DM alters the regulation of glucose over multiple scales of time. To test this hypothesis, we have introduced a novel analytical approach termed Multi-Scale glycemic variability (Multi-Scale GV), which is based upon the Ensemble Empirical Mode Decomposition (EEMD) algorithm [22]–[24]. This approach, which has no priori assumptions regarding cycle length, signal linearity or stationarity, enables identification of the degree of glycemic variability for multiple unique oscillatory cycles with average periods ranging from minutes to hours. We have compared Multi-Scale GV measures to traditional measures of glycemic variability to validate this approach. Further, we analyzed glycemic variability for entire recording period, but also separately for day and night periods to determine whether day-time behaviors affect glycemic variability at specific time-scales. We further hypothesized that: 2) Increases in glycemic variability at specific time-scales would be related to structural changes in central autonomic network and worse cognitive function. Therefore, we sought the relationships among glycemic variability and gray matter volumes in central autonomic network, and cognition. Finally, we hypothesized that: 3) In type 2 DM, observed relationships between glycemic variability, brain structure and cognitive function would be independent of long-term glycemic control, e.g., hemoglobin A1C (HbA1c) and the prevalence of hypoglycemic episodes (<70 mg/dL). Hence, we studied the relationship between glycemic variability, brain structure and cognitive function in older adults with type 2 DM and age-matched controls.

## Research Design and Methods

### Ethics Statement

Experiments were conducted in the Syncope and Falls in the Elderly Laboratory at the Clinical Research Center and the Center for Advance Magnetic Resonance Imaging (MRI) at Beth Israel Deaconess Medical Center (BIDMC). Participants were recruited consecutively and provided informed written consent as approved by the Institutional Review Board (IRB, i.e., the Committee on Clinical Investigations, Beth Israel Deaconess Medical Center). The study has been approved by BIDMC IRB and consent forms are available upon request.

### Subjects

The cohort consisted of 43 volunteers with type 2 DM aged 50–85 years, and 26 non-diabetic, age-, sex- and cardiovascular risk-matched adults with normal fasting glucose and HbA1c. Recruitment was completed via community advertisement. Subjects in DM group were diagnosed with type 2 DM and treated >5 years with oral agents and/or combinations with insulin, either normotensive (BP<140/90 mm Hg and no medical history of hypertension) or hypertensive (BP>140/90 mm Hg and/or treated for hypertension). Control subjects were – normal glucose, HBA1c, MMSE, with and without hypertension.

Type 2 DM subjects were treated with insulin (11), oral glucose-control agents (sulfonylurea, second generation agents or their combinations (34)), or diet (5), and for hypertension (7) and hyperlipidemia (27). Controls were treated for hypertension (9) and/or hyperlipidemia (11).

Exclusion criteria were: type 1 DM, history of stroke, subacute myocardial infarction, significant cardiac diseases, arrhythmias and nephropathy, kidney or liver transplant, congestive heart failure, carotid artery stenosis, neurological or other systemic disorders, dementia or sub-threshold Mini Mental Status Exam (MMSE) scores (≥3 points below the comparative normal value for the subject's age group and education level, or ≤24), current recreational drug or alcohol abuse, morbid obesity (BMI≥40), claustrophobia, or 3T MRI-incompatible metal implants, pacemakers or arterial stents.

Of 130 participants (including 74 type 2 DM subjects and 56 control subjects), 30 type 2 DM subjects and 26 control subjects were excluded. Besides, 5 eligible subjects were also excluded from the analysis due to incomplete continuous glucose monitoring (CGM) recordings.

### Protocol

CGM and 24-hour ambulatory blood pressure monitoring (ABPM) [25] were assessed for three days prior to an overnight admission to the BIDMC Clinical Research Center. A nurse set up CGM and also trained participants to manually measure their BP and glucose, four times daily (i.e., before meals and at bed time), using arm cuff and finger stick methods. Participants were directed to go to bed at 10pm, wake at 7am and maintain their typical activities. They documented sleep/wake times, meals and medications in a diary. Upon admission, neurological and neuropsychological assessments were performed. A fasting blood draw and urinary sample were obtained the next morning for routine glucose, lipid and renal panels. The MRI was then completed.

### Continuous Glucose Monitoring

The iPro Professional CGM (Medtronic, Minneapolis MN) enabled multi-day glucose measurements with a waist-level subcutaneous sensor [26]. The sensor obtained interstitial glucose levels every five minutes and was calibrated four times daily via finger stick [27].

### Neuropsychological Measures

Neuropsychological assessments included the Mini-Mental State Examination (MMSE), Hopkins Verbal Learning Test–Revised (HVLT, verbal learning and memory function, including a Total Recall (total number of list items learned across trials), Delayed Recall (total number of list items recalled after the delay), Retention (percentage of items from Total Recall that are subsequently recalled on Delayed Recall), and Recognition Discrimination Index (number of list items correctly identified among non-list items)) [28], Rey-Osterreith Complex Figure Test (ROCFT, a measure of visual-spatial ability and visual memory function, including Immediate Recall and Delayed Recall), Trail Making Test A and B (TMT, executive function), and Verbal Fluency (a measure of executive function, dependent variables for the fluency measures include number of items generated for each of three phonemic trials (e.g., F, A, S) and the number of items generated for the semantic task (e.g., animals)) [29]. The Geriatric Depression Scale (GDS) and the Instrumental Activities of Daily Living (IADL) Scale were also completed. The neuropsychological test results were analyzed using age-adjusted standardized T scores.

Composite cognitive performance for learning and memory function (“learning and memory T score”) was calculated as an average of HVLT and ROCFT T scores. Composite cognitive performance for executive function (“executive function T score”) was calculated as an average of Verbal Fluency and TMT T scores. A total composite cognitive function (“Composite T score”) was calculated as the average of all the T scores [30]. All the T scores were adjusted for age, sex, race and educational levels.

### Magnetic Resonance Imaging

Studies were performed on a 3-Tesla GE GHX MRI scanner using a quadrature and eight-channel phase array head coils (GE Medical Systems, Milwaukee, WI). Anatomical images were acquired using 3-D magnetization prepared rapid gradient echo (MP-RAGE) and fluid attenuated inversion recovery (FLAIR) sequences. Images were analyzed using tools developed in interactive data language (IDL, Research Systems, Boulder, Colorado, USA) and MATLAB (MathWorks, Natick, Massachusetts, USA). Anatomical MR images (MP-RAGE and FLAIR) were co-registered non-linearly to the MNI152 standard template and segmented to calculate regional gray and white matter and cerebrospinal fluid volumes in main anatomical lobes and their sub-regions (SPM, University College London, UK) [31]. Sub-regions were defined according to the LONI Probabilistic Brain Atlas (LPBA40). Each lobe (e.g. frontal, temporal etc.) was divided according to anatomical divisions and structures (e.g. middle orbitofrontal gyrus, superior frontal gyrus, hippocampus, etc.).

### Glycemic Variability Measurements

Glycemic variability was calculated from the entire 72-hour CGM time-series. Traditional metrics included the standard deviation (SD), mean glycemic excursions (MAGE) [32] and the number and duration of hypoglycemic episodes defined as glycemic levels <70 mg/dL. MAGE was calculated as the arithmetic mean of glucose increases or decreases (from glucose nadirs to peaks or vice versa) when both ascending and descending segments exceeded the value of one SD of mean glucose [32].

To study glycemic variability at multiple temporal scales, we propose a new measurement called Multi-Scale Glycemic Variability (Multi-Scale GV). This technique decomposes the original CGM time-series using EEMD. This adaptive data analysis technique automatically identifies periodicities intrinsic to the time-series, which underlie its fluctuations at different time-scales. It does not assume linearity or stationarity of the series, thereby offering advantages over traditional approaches such as Fast Fourier Transform (FFT) or wavelet decomposition [33], [34]. Each CGM time-series was decomposed into multiple new time-series, termed intrinsic mode functions (IMFs), characterized by a dominant frequency band. We refer to each IMF as a glycemic variability cycle (GVC). Thus, Multi-Scale GV enables the identification and subsequent quantification of GVCs at multiple time scales from minutes to hours without any priori assumptions of cycle duration.

### Multi-Scale Glycemic Variability Measurement based on EEMD Algorithm

Computational Steps for Multi-Scale GV analyses are described as follows:

#### Step 1

*Decompose the raw CGM data (72 hours) of each subject into IMFs, here referred as glycemic variability cycles (GVCs).* Each GVC consists of narrow-band frequency-amplitude modulations. The GVCs were obtained via EEMD, which is a noise-assisted improvement of the EMD method [22], [23], [35], [36], briefly introduced in ***Text S1***.

#### Step 2

*Calculate the average period (time-scale) of each GVC. As CGM sampling frequency was 5 minutes and sampling duration was 72 hours, we selected the GVCs with period less than 24 hours*. GVCs are non-stationary, with varying amplitude and frequency. The calculation of the average period for each GVC is shown in ***Text S2***.

#### Step 3

*For each subject, quantify the glycemic variability at multiple cycles (or time-scales) by calculating the standard deviation for each GVC obtained in* ***Step 2*** *.*

### Statistical Analysis

All variables were summarized using descriptive statistics and compared between groups using one-way ANOVA and non-parametric tests.

To test our first hypothesis that type 2 DM alters the regulation of glucose over multiple scales of time, we compared all markers of glycemic variability between groups using one-way unadjusted ANOVAs, and between day and night using two-way ANOVA.

To compare Multi-Scale GV to traditional glycemic measures (SD and MAGE), we used least square models adjusted for age, sex and group. Receiver operating curves (ROC) were used to compare the sensitivity and specificity of Multi-Scale GV, SD and MAGE.

To test our second hypothesis that increases in glycemic variability at specific time-scales would be associated with brain tissue atrophy and cognitive function, we first adjusted cognitive test scores for age, sex and education. A correlation matrix was initially used to determine associations among multiple variables (age, sex, regional MR volumes and cognitive). Those correlations with *r^2^*>0.1 and *P*<0.05 were included in the modeling approach. Least squares models were then used to assess the relationships between glycemic variability and outcome measures. Independent variables were GVCs, SD and MAGE, and dependent variables were regional brain volumes and cognitive functional measures. These models were calculated for each parameter separately to minimize repeated measures effects. In results section, we presented *r^2^* adjusted for co-variants. For the relationship between Multi-Scale GV and brain volumes, we conservatively selected only models with adjusted *r^2^*>0.25, and *P*<0.05 from models adjusted for age, sex, group.

To test our third hypothesis that observed relationships between glycemic variability, brain structure and cognitive function would be independent of long-term glycemic control (i.e., fasting glucose, HbA1c, DM duration, hypoglycemic episodes), least squares models were first used to assess the relationships between HbA1c (or hypoglycemic episodes), brain volumes and functional outcomes, then used to assess the relationship between glycemic variability, brain volumes and functional outcomes. Models were adjusted for age, sex, group, glucose, HbA1c and hypoglycemic episodes. We also examined the relationship between HbA1c, SD, MAGE and brain volumes, functional outcomes, using least squares models adjusted for age, sex and group.

The effects of diabetes duration, medication classes, BP, hypertension, HbA1c, body mass index and other confounders were also evaluated.

All the relationships were assessed using least squares models adjusted for age and sex (and group for all subjects), and we presented *r^2^* for the entire model and *P* values for the specific effect of Multi-scale GV.

## Results

### Characteristics of the Study Cohort

Table 1 summarizes cohort demographics, cardiovascular and metabolic outcomes, cognition and global brain volumes. There were no between-group differences in age, sex, education, 24 hour systolic and diastolic BP, reduction of diastolic BP during sleep, average number or duration of hypoglycemic events, depression, Trail Making Test, MMSE score, or global CSF. As compared to controls, the type 2 DM group had lower body mass index, HbA1c and fasting glucose. This group also exhibited worse composite cognitive function T scores, lower HVLT and ROCFT performance (indicating impaired learning and memory).

**Table 1:** Characteristics of the study cohort.

	Diabetes (n = 43)	Control (n = 26)	*P*-value
*Demographics*			
**Age (years)**	65.52±8.73	65.21±10.23	0.89
**Sex (male, female)**	20, 23	14, 12	0.56
**Body mass index (kg/m^2^)**	29.92±4.90	25.20±4.63	<0.0001
**Race (W, AA, Latino, other)**	28, 12, 2, 1	25,1, 0, 0	0.032
**Education (years)**	15.17±3.62	15.92±3.02	0.33
*Cardiovascular and Metabolic Outcomes*			
**Hypertension (yes/no)**	35/43	7/26	<0.0001
**24 hour systolic BP (mmHg)**	131.21±9.47	126.92±8.50	0.08
**24 hour diastolic BP (mmHg)**	67.73±7.31	67.40±8.81	0.89
**Microalbumin, urine (ug/mL)**	11.94±16.35	19.16±33.22	0.56
**Cholesterol-to-HDL ratio**	3.80±2.64	3.18±1.03	0.34
**Triglycerides (mg/dL)**	139.23±87.01	105.30±59.02	0.03
**Total number of Hypoglycemic Events**	1.80±2.80	4.00±5.80	0.29
**Average duration of Hypoglycemic Events (min)**	46.45±30.75	53.69±33.76	0.52
**Diabetes duration (years)**	13.33±6.81	-	-
**Hematocrit (%)**	39.80±8.23	40.16±3.62	0.06
**Hemoglobin A1C (%)**	7.16±1.17	5.66±0.29	<0.0001
**Hemoglobin A1C (mmol/mol)**	54.7±12.8	38.2±3.2	<0.0001
**Fasting Glucose (mg/dL)**	125.67±47.32	89.83±10.09	0.0002
*Cognitive Outcomes*			
**Composite “Learning and Memory T score”**	43.44±9.40	51.35±9.18	0.0004
- Hopkins Verbal Learning (T Score)	44.16±9.44	52.65±7.93	<0.0001
- Rey-Osterrieth Complex Figure Test (T score)	42.26±11.22	48.39±13.43	0.03
**Composite “Executive function T score”**	40.84±7.60	49.75±7.70	<0.0001
- Verbal Fluency (T score)	37.22±7.72	51.61±9.26	<0.0001
- Trail Making Test (T score)	44.56±9.93	47.78±9.07	0.15
**Overall “Composite T score”**	42.20±7.06	51.43±7.27	<0.0001
**MMSE Scores (range: 25–30)**	28.46±1.54	29.00±1.52	0.17
**Instrumental Activities of Daily Living**	26.19±1.31	26.00±3.04	0.74
**Geriatric Depression Scale**	7.12±1.14	4.83±1.39	0.21
*Global Brain Volumes (cm^3^)*			
**Gray matter**	622.16±72.96	666.62±74.83	0.02
**White matter**	422.63±57.44	461.43±57.81	0.01
**Cerebrospinal Fluid**	611.78±206.53	647.51±211.05	0.51

Data are presented as mean ± standard deviation (SD). P values were obtained by One-Way ANOVA to compare group means and using Wilcoxon Test for not normally distributed variables. The variables analyzed using Wilcoxon Test are Age, Sex, Race, Education, Hypertension, Microalbumin (urine), Cholesterol-to-HDL ratio, Triglycerides, Total number of Hypoglycemic Events, Average duration of Hypoglycemic Events, and Hematocrit, and other variables were analyzed using One-Way ANOVA. MMSE: Mini-Mental State Examination.

### Multi-Scale Glycemic Variability (Multi-Scale GV)

**Figure 1:**
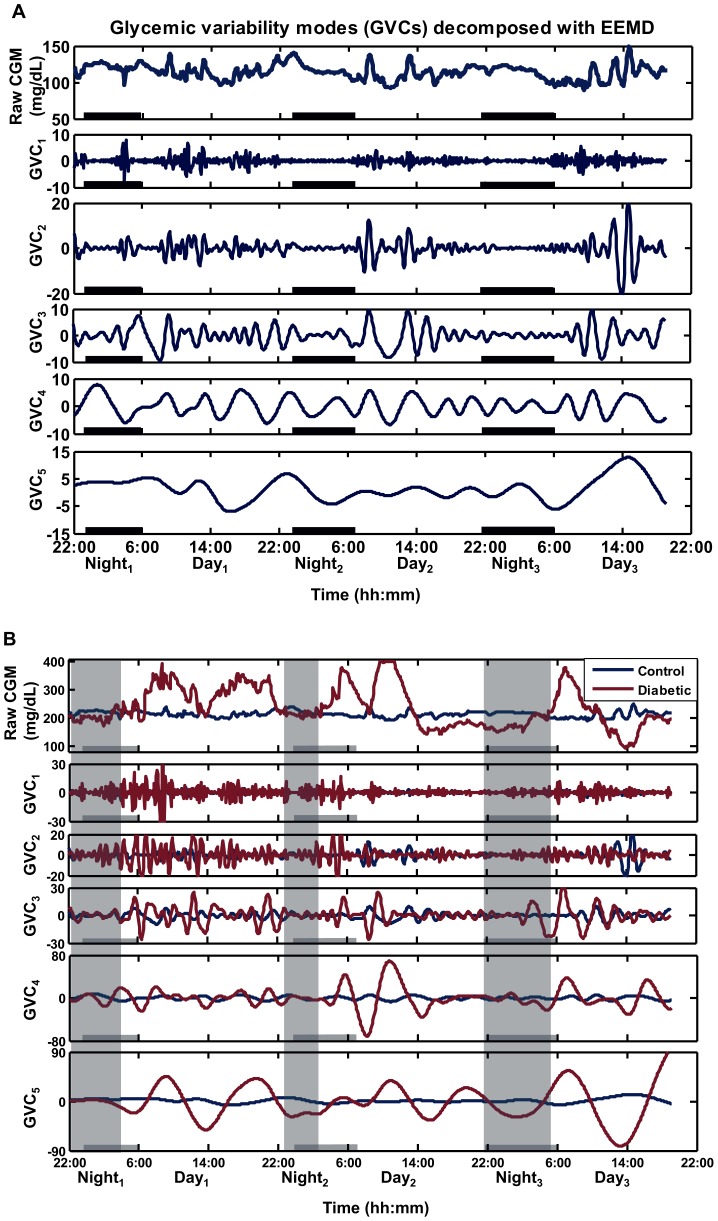
The Multi-Scale Glycemic Variability method applied to 3-day continuous glucose monitoring (CGM). The decomposition is based on the EEMD (Ensemble Empirical Mode Decomposition) technique. (A) The original CGM time-series from a representative control subject (male, 72 years old, HbA1c = 5.2%, SD (standard deviation) = 10.44, MAGE (mean average glycemic excursions) = 31.61) is decomposed into five glycemic variability cycles (GVCs) that are each characterized by fluctuations within a specific frequency band. The bold black lines along the X-axis denote sleep periods defined by actigraphy and patient records. (B) Comparison of raw CGM signals and selected GVCs between the control subject in (A) and a representative patient with type2 DM (male, 62 years old, HbA1c = 9.4%, SD = 71.36, MAGE = 161.85). The shading areas denote sleep for the type 2 DM patient, while the bold black lines along the X-axis denote sleep for the control subject.

Multi-Scale GV identified serum glucose fluctuations at five unique frequencies in type 2 DM subjects and controls: GVC_1_ (∼0.5 hour), GVC_2_ (∼1 hour), GVC_3_ (∼2 hours), GVC_4_ (4–5 hours), GVC_5_ (9–12 hours) (Table 2). The period of GVC_4_ (type 2 DM: 4.75±1.14 hours; controls: 3.84±1.05 hours) coincides with meal cycles as reported in home diaries (type 2 DM: 4.41±1.17 hours; controls: 3.99±1.35 hours) and the period of GVC_5_ (9–12 hours) may reflect sleep/wake cycle. Fig. 1 provides an example of this technique as applied to the data of a representative control subject alone (Fig. 1A), as well as in comparison with an age-matched patient with type 2 DM (Fig. 1B).

**Table 2:** Comparisons of glycemic measures and glycemic variability measures between diabetic and control groups y.

Glycemic variability (mg/dL)			Diabetics (n = 43)	Controls (n = 26)	*P*-value
**Home monitoring-finger stick glucose**			148.06±34.92	102.18±10.04	<0.0001
**SD of glucose finger stick measurements**			44.99±28.84	14.66±5.48	<0.0001
**Mean CGM glucose value**			150.14±32.52	107.97±16.91	<0.0001
**SD (Standard deviation)**			38.39±21.73	17.44±8.12	<0.0001
**MAGE (Mean Glycemic Excursions)**			95.22±55.39	39.45±16.49	<0.0001
**Multi-Scale GV**	**GVC_1_**	**period (hour)**	0.63±0.03	0.62±0.07	0.51
		**Variability**	2.62±1.63	1.99±1.01	0.08
	**GVC_2_**	**period (hour)**	0.86±0.12	0.90±0.07	0.1
		**Variability**	4.53±2.3	3.52±1.62	0.055
	**GVC_3_**	**period (hour)**	1.92±0.41	1.92±0.17	0.99
		**Variability**	8.56±3.04	4.83±2.45	<0.0001
	**GVC_4_**	**period (hour)**	4.75±1.14	3.84±1.05	0.004
		**Variability**	14.76±7.68	5.72±3.03	<0.0001
	**GVC_5_**	**period (hour)**	11.30±2.38	8.54±2.73	<0.0001
		**Variability**	19.48±12.56	6.68±3.66	<0.0001

Data are presented as mean ± SD and were calculated from 72 hour recordings. *P* values were obtained by One-Way ANOVA. SD: standard deviation; CGM: continuous glucose monitoring; Multi-Scale GV: Multi-Scale glycemic variability; GVC: glycemic variability cycle.

**Figure 2:**
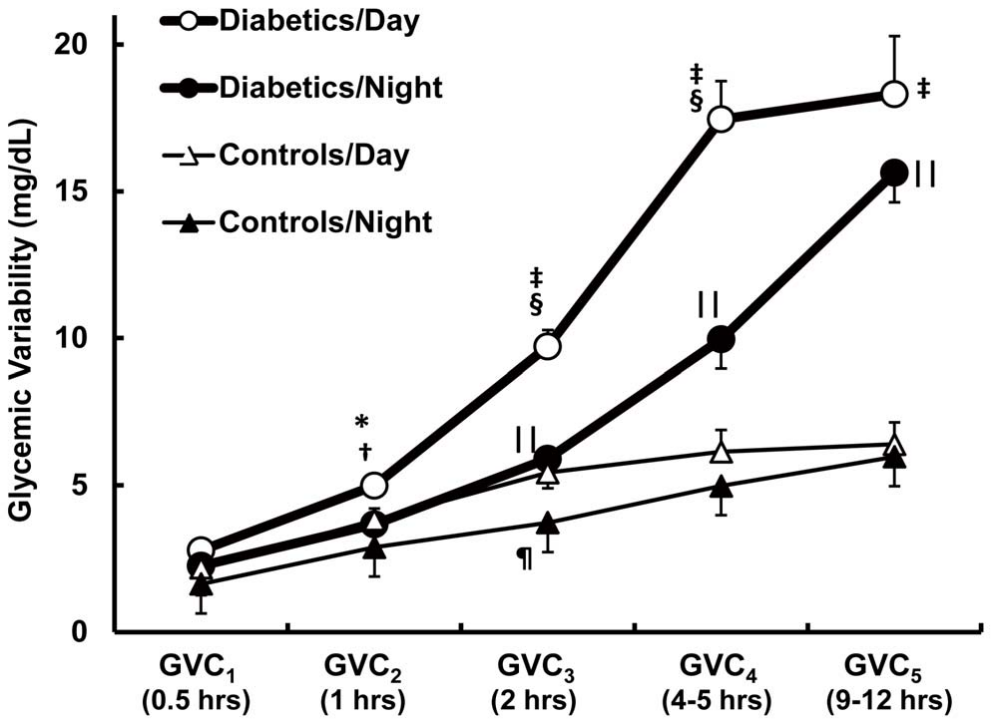
Day and night Multi-Scale Glycemic Variability in older adults with and without type 2 DM As compared to controls, the type 2 DM group had greater variability during the day in GVC_2–5_, and night GVC_3–5_. At night, glycemic variability declined in type 2 DM in GVC_2–4_ and in controls in GVC_3_. ‘*’ (*P* = 0.002) and ‘‡’ (*P*<0.0001) indicate significant differences between diabetics/day and controls/day; ‘∥∥’ (*P*<0.0001) indicates significant differences between diabetics/night and controls/night; ‘†’ (*P* = 0.003) and ‘§’ (*P*<0.0001) indicates significant differences between diabetics/day and diabetics/night; ‘¶’ (*P* = 0.028) indicates significant difference between control/day and control/night. All the *P* values were obtained by ANOVA. Results are presented as mean ± SEM.

As compared to controls, the type 2 DM group had greater variability in GVC_3–5_ (*P*<0.0001) and longer cycle durations in GVC_4_ (*P* = 0.004) and GVC_5_ (*P*<0.0001). We also separately analyzed the effects of sleep/wake behavior on glycemic variability (Fig. 2). GVC_1–5_ was identified during both day and night in both groups, but the variability was greater in the DM group. Type 2 DM subjects had greater variability than controls both during the day (GVC_2–5_, *P* = 0.00001–0.031) and night (GVC_3–5_, *P*<0.0001). In the type 2 DM group, variability was less at night as compared to day (GVC_2_, *P* = 0.002; GVC_3–4_, *P*<0.0001). In controls, variability was less at night as compared to day only in GVC_3_ (*P* = 0.028).

### Relationships between Multi-Scale GV, Conventional Markers of Glycemic Variability and Long-Term Glycemic Control

All conventional markers of glycemic variability were greater in diabetic subjects as compared to controls (Table 2) (e.g. SD, MAGE of CGM data, and mean, SD of glucose values obtained from subject-recorded finger sticks) (*P*<0.0001).

**Figure 3:**
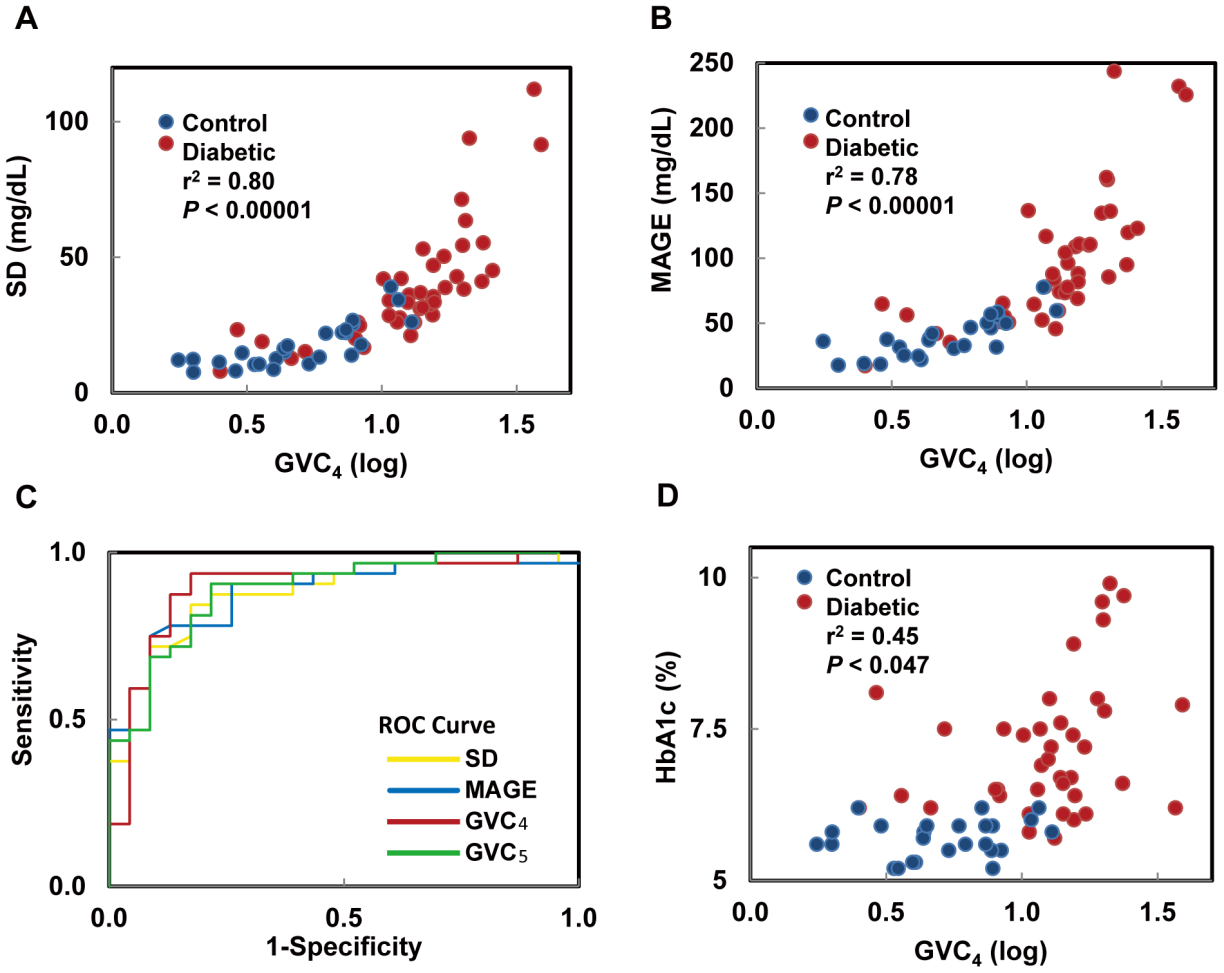
Relationships between the fourth glycemic variability cycle (GVC_4_) and conventional measures of glycemic control. The degree of glycemic variability within GVC_4_ was highly correlated with SD (A) and MAGE (B), but the areas under the curves of GVC_4_ and GVC_5_ were greater than SD and MAGE (C). The degree of glycemic variability within GVC_4_ was highly correlated several markers of glucose control including HbA1c (D). As with GVC_4_ (the cycle linked with meal intake), the example in this figure, similar relationships were observed for all other GVC cycles. The *r^2^* and *P* values represent the least square model fit.

The degree of variability within each GVC correlated with SD of CGM (*r^2^* = 0.46–0.89, *P*<0.005) (Fig. 3A), MAGE (*r^2^* = 0.50–0.90, *P*<0.0004) (Fig. 3B) and both the average and SD of glucose values measured by finger sticks during home monitoring (*r^2^* = 0.32–0.62, *P*<0.01). However, using an area under the curve (ROC) analysis, GVC_4–5_ performed better at classifying diabetic and control subjects (larger area and higher sensitivity and specificity) than either SD or MAGE (Fig. 3C).

Across all subjects, greater variability within one or more GVCs was also associated with worse glycemic control, including higher fasting glucose (*r^2^* = 0.31–0.34, GVC_1–5_ *P* = 0.0006–0.036), higher HbA1c (*r^2^* = 0.45–0.50, *P*<0.05) (Fig. 3D). In the type 2 DM group, greater variability within GVC_5_ was associated with longer type 2 DM duration (*r^2^* = 0.26, *P* = 0.033), however, no measure of glycemic variability correlated with the number or duration of hypoglycemic episodes (defined as glycemic levels <70 mg/dL).

### Relationship between Multi-Scale GV and Brain Volumes

**Figure 4:**
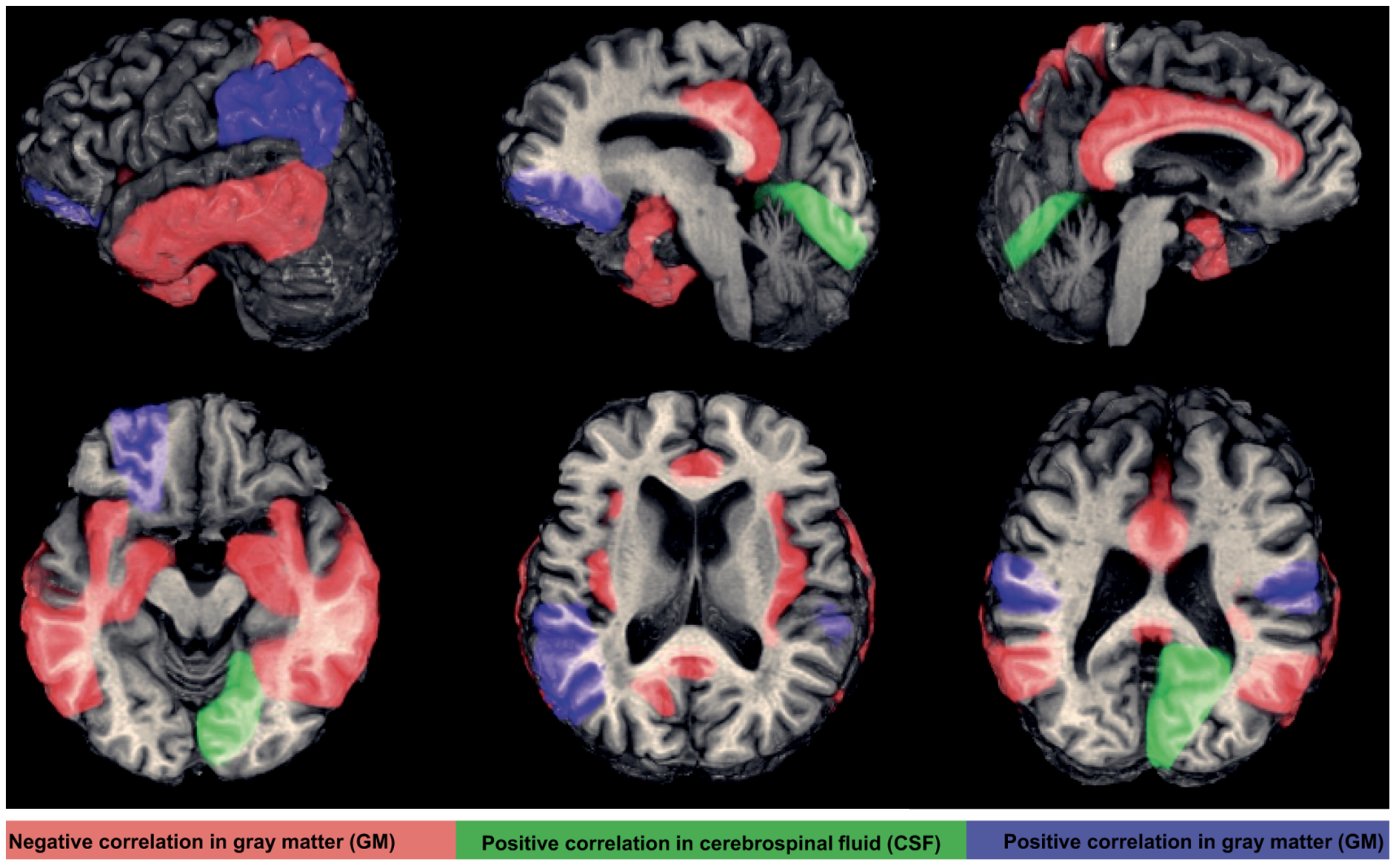
The brain regions associated with Multi-Scale GV. Higher glycemic variability of GVC_1–3_ (period 0.5–2 hours) were associated with lower gray matter (GM) volume (red color; both hemispheres in the cingulate gyrus, hippocampal gyrus, middle and inferior temporal gyrus, insular cortex, the left superior parietal gyrus and right fusiform gyrus), greater GM volume (blue color; the bilateral supramarginal gyrus, left angular gyrus and left middle orbitofrontal gyrus), and greater cerebrospinal fluid (CSF) in the right lingual gyrus (green color).

**Figure 5:**
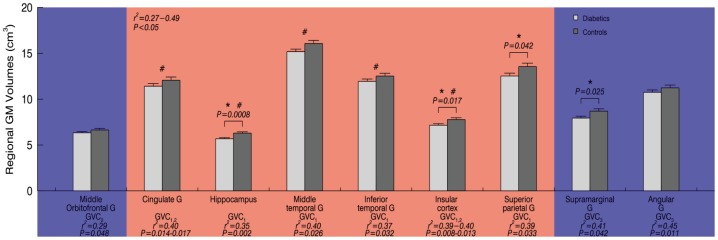
Group differences of regional GM volumes in left hemisphere and their relationship with Multi-Scale GV. ‘*’ indicates significant differences between the type 2 DM group (white) and controls (grey) in GM volumes (One-Way ANOVA); regional GM volumes in left hemisphere were correlated with Multi-Scale GV for diabetics and/or controls, blue indicates positive correlation, red indicates negative correlation with each GVC, G' = gyrus, ‘#’ indicates we found similar relationship between Multi-Scale GV and GM volumes in the right hemisphere (*r^2^* = 0.26–074, *P*<0.05). The bar graphs are presented as mean ± SEM.

As compared to controls, subjects with type 2 DM had lower global gray matter (GM, *P* = 0.02) and white matter (WM, *P* = 0.01) volumes, but not cerebrospinal fluid (CSF) volume (Table 1). Regionally, diabetic subjects exhibited reduced GM within the hippocampus, insular cortex, superior parietal gyri and supramarginal gyri (*P*<0.05) (Fig. 4, and Fig. 5).

The presented relationships between Multi-Scale GV and regional brain volumes were got from least squares models for the entire cohort, as well as within each group separately.

**Figure 6:**
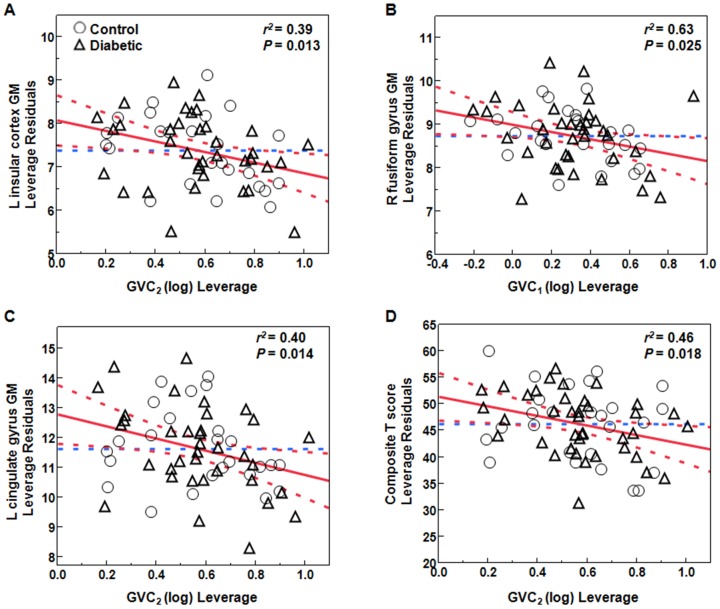
Examples of least squares models indicating negative relationships between Multi-Scale GV and regional GM volumes as well as cognitive performance. (A) relationship between GVC_2_ and GM volume in the left insular cortex; (B) relationship between GVC_1_ and GM volume in the right fusiform gyrus; (C) relationship between GVC_2_ and GM volume in the left cingulate gyrus; (D) relationship between GVC_2_ and overall cognitive performance (composite T score) (diabetics: triangles; controls: circles). We presented *r^2^* for the entire model adjusted for age and sex and group, and *P* values for the specific effect of Multi-scale GV.

Across all subjects, greater glycemic variability within higher frequency cycles (GVC_1–2_, 0.5–1 hours) was associated with lower GM volumes (*r^2^* = 0.26–0.74, *P* = 0.001–0.048) and more CSF volume (*r^2^* = 0.25–0.50, *P*<0.05) in multiple regions within the limbic system, as well as several temporal and parietal regions linked to learning and memory, e.g. bilaterally within the cingulate gyrus, hippocampus, middle temporal gyrus, inferior temporal gyrus and insular cortex, as well as within the left superior parietal gyrus and right fusiform gyrus (Fig. 4, Fig. 5 and Fig. 6).

In the type 2 DM group only, greater variability in GVC_2_ was correlated with lower GM volumes within the cingulate gyrus (*r^2^* = 0.40, *P* = 0.02) and insular cortex (*r^2^* = 0.33, *P* = 0.032). However, we also found that higher glycemic variability of GVC_3_ associated with greater GM in supramarginal gyrus and angular gyrus (*r^2^* = 0.41–0.45, *P* = 0.01–0.04).

In the control group only, the degree of variability within GVC_3–5_ associated with more CSF within the hippocampal gyrus (*r^2^* = 0.40–0.48, *P*<0.02) and lingual gyrus (*r^2^* = 0.44–0.47, *P* = 0.02–0.04). Greater variability of GVC_5_ was associated with larger GM volume of the subcortical motor nuclei (caudate nucleus, *r^2^* = 0.30–0.33, *P*<0.03). However, we also found that higher glycemic variability of GVC_3_ associated with greater GM in middle orbitofrontal gyrus (*r^2^* = 0.29, *P* = 0.048).

HbA1c, fasting glucose, SD and MAGE were not independently associated with brain volumes and functional outcomes after controlling for GVC variability measures.

### Relationship between Multi-Scale GV and Cognitive Function

The presented relationships were for the entire cohort, as well as for DM group and control group separately.

In the entire cohort, greater glycemic variability in GVC_2,3,5_ was associated with worse learning and memory function (i.e. HVLT T score, ROCFT T score, and the composite “learning and memory T score”, *r^2^* = 0.27–0.34, *P* = 0.011–0.036). Greater glycemic variability in GVC_2,5_ was associated with worse overall composite cognitive performance (i.e. “Composite T score”, *r^2^* = 0.45–0.46, *P*<0.02). T scores related to executive function measures, such as TMT T score, verbal fluency T score, and “executive function T score”, were not significantly correlated with Multi-Scale GV.

In type 2 DM group only, greater glycemic variability in GVC_2,3_ was associated with worse learning and memory function (i.e. “learning and memory T score”, *r^2^* = 0.28–0.37, *P*<0.03), greater variability in GVC_2_ was also associated with worse overall cognitive performance (i.e. “Composite T score”, *r^2^* = 0.44, *P* = 0.02) and greater variability in GVC_5_ was associated with more depression (i.e. GDS, *r^2^* = 0.18, *P* = 0.014).

No significant relationships between Multi-Scale GV and cognitive function were found in control group (*r^2^*<0.13, *P*>0.05).

We also analyzed nonparametric multivariate correlations among glycemic variability, gray matter atrophy and cognition. Across all subjects, greater variability of GVC_2_ was associated with both lower GM volume in cingulate gyrus (*r^2^* = 0.38–0.40, *P* = 0.014–0.047) and worse cognitive performance (i.e. “Composite T score”, *r^2^* = 0.46, *P* = 0.018) (Fig. 6 C and D), and worse scores on HVLT (*r^2^* = 0.42–0.46, *P* = 0.002–0.006). Combined effects of greater variability in GVC_2_, lower GM in cingulate gyrus and longer DM duration were highly associated with more depression (*r^2^* = 0.69, *P*<0.02).

## Discussion

This study evaluated the complex relationships between glycemic variability, brain volumes and functional outcomes in older adults with and without type 2 DM. It determined that time-specific glycemic fluctuations may be independently associated to brain atrophy and worse cognitive performance.

We applied a novel EEMD-based approach to quantify glycemic variability at multiple time scales. The EEMD method identifies dominant cycles without a priori assumptions about their periods and is therefore suitable for nonlinear and non-stationary signals such as continuous monitoring of glycemic levels monitoring. Multi-Scale GV determined that glucose levels fluctuate at five distinct frequencies (GVC_1–5;_ period 0.5 to 12 hours) in both diabetics and controls. The GVCs frequencies coincide with one or more ultradian rhythms that modulate glucose levels. For example, the periods of GVC_1–3_ (0.5–2 hrs) correspond to established autonomic rhythms, cardiovascular and neuroendocrine rhythms (insulin and glucocorticoids) [13]–[17] and certain aspects of the sleep cycle (i.e. rapid and non-rapid eye movement) [12]. The period of GVC_4_ (4–5 hours)) coincides with meal cycles as reported in home diaries and the period of GVC_5_ (9–12 hours) may reflect sleep/wake cycle. Type 2 DM subjects had greater glycemic variability as compared to controls in GVC_3–5_, as well as longer cycles that tended to be even lengthier in type 2 DM subjects with higher HbA1c levels.

To resolve the complex effects of glycemic variability on brain volumes and cognitive function, we studied the relationship between Multi-Scale GV, regional brain volumes and cognition. We observed that GVCs at specific frequencies were related to different regional brain volumes and functional outcomes, and that these relationships were independent of HbA1c, hypoglycemic episodes, age and sex.

Glucose metabolism is closely coupled with the autonomic nervous system via oscillating neural networks and particularly beta-adrenergic and cholinergic systems in the brain, controlling regional perfusion during spontaneous brain activity, cognitive and motor tasks. The complex interplay between glucose, neuroendocrine and autonomic rhythms is controlled by a system comprising a central pattern generator (suprachiasmatic nucleus of the hypothalamus, SCN) and central autonomic network through complex feedback mechanisms insulin secretion, glucose absorption and energy expenditure [37], [38].

Greater variability in GVC_1–3_ (0.5–2.0 hours) were strongly associated with gray matter atrophy affecting the limbic system, insular cortex, hippocampus and cognitive and visuospatial orientation circuits within the temporal and parietal lobes. These associations are clinically relevant because they indicate a link between glycemic variability and altered central autonomic regulation that translates into adverse functional outcomes. These regions belong to the limbic system and central autonomic networks, also known as the “central command” to the sympathetic and parasympathetic control of heart rate and blood pressure, gastrointestinal system etc., as well as, selective attention and emotional arousal processes, and mood. Greater variability in GVC_1–3_ was associated with lower gray matter volume in circuits for learning and controlling memory (cingulate gyrus, hippocampus), visuospatial processing (superior parietal and fusiform gyrus) motor function and gait (inferior temporal gyrus). In type 2 DM group specially, greater glycemic variability in GVC_2–3_ was associated with worse learning and memory. In contrast, positive relationships between gray matter and glycemic variability in controls may suggest regional compensatory activity needed to maintain glycemic variability in a dynamic stable range.

There is also evidence that brain atrophy is accelerated by type 2 DM [3], [4]. Such atrophy manifests preferentially within fronto-temporo-parietal regions and is associated with cognition and gait abnormalities [39]. We report that glycemic fluctuations at specific frequencies, yet not hypoglycemic episodes, have distinct effects on gray matter volumes in specific regions. Traditional measures such as hypoglycemic episodes, HbA1c, SD and MAGE did not correlate with brain atrophy. In addition, separating day and night time, the associations between Multi-Scale GV and brain volumes were stronger at night than during day.

The low frequency GVC_4_ coincided with food intake (∼4–5 hours) as confirmed by home diaries. Yet, GVC_4_ fluctuations were also present during the night, suggesting an underlying intrinsic regulation of eating patterns. Variability in GVC_4_ increased with higher HbA1c and correlated with SD and MAGE calculated from CGM. Sleep/wake behavior is reflected in GVC_5_ mode (∼9–12 hours). Notably in diabetics, greater variability in GVC_5_ was associated with longer DM duration and more depression. Multi-Scale GV was more sensitive and specific than SD and MAGE, indicating that these traditional measures may not capture the complex impacts of variability that occurs at specific time scales. An observational study suggested that reduction in SD and MAGE was associated with better glycemic control without increase in hypoglycemic events [40]. In the present study, hypoglycemic episodes, which are commonly viewed as a barrier to successful glycemic control, were not associated with specific GVCs, brain atrophy or functional abnormalities.

Our findings suggest that Multi-Scale GV in type 2 DM is associated with worse learning and memory functions but not with executive functions. These results are consistent with population based studies that identified type 2 DM affects specific cognitive abilities, namely processing speed and specific types of memory [41], [42]. Type 2 DM associated with slower processing speed and impaired semantic memory, but not with episodic or working memory [41], suggesting cognition may reflect a vascular process combined with insulin resistance in the brain. Our composite measure of memory reflects verbal and visual-spatial memory. Hyperglycemia-induced insulin resistance and small vessel disease is a common pathway for abnormal blood brain barrier (function, neurovascular coupling, regional vasoreactivity and hypoperfusion [43]–[45] and neurotoxicity [46] and neuron-astrocyte signaling), which are key processes involved in memory formation. However, the exact mechanisms by which type 2 DM leads to cognitive decline requires further investigation, notably because a strict glycemic control did not improve cognitive function (ACCORD-MIND) in a population study. Indeed, type 2 DM is associated with an increased risk of both vascular dementia and Alzheimer's disease [47], although there is evidence suggesting that the association may be stronger for Alzheimer's disease with cerebrovascular disease.

There was no association between cognitive performance and glycemic variability in control group, while in the type 2 DM group there was an association. This might be because of the differences in cognitive function of the two groups. The lower cognitive function of those with diabetes might be suggestive of incipient dementia. The stronger association between cognition and glycemic variability may be due to subjects with type 2 DM having a greater variety of pathologies in addition to the neural differences. For example, cardiovascular diseases are more prevalent in persons with diabetes. Glycemic variability was associated with memory but not with executive functions, which has been consistently associated with cerebrovascular disease.

Multi-Scale GV allows us to overcome limitations of traditional measures and to study effects of glycemic variability at specific frequencies, and to thus better understand this phenomenon. Multi-Scale GV provides information about physiological rhythms that modulate serum glucose at specific time scales and affect signaling among multiple organ systems. Our current observations suggest that GVCs at multiple time-scales may be related to intrinsic rhythms, generated by central pattern generator(s), and also modulated by behavior factors (meals and/or sleep). Diabetes alters the cycle length and amplitude of these oscillations, as well as their relationship to physiological rhythms. These long-term alterations in brain structural networks appear to manifest as functional decline. Therefore, this novel approach has indicated that variability associated with several ultradian rhythms is linked to regional brain atrophy and worse functional measures, independent of HbA1c levels and hypoglycemic episodes. This study thus provides support for the use of Multi-Scale GV to understand, monitor and develop diagnostics and therapeutics for treating glycemic variability independently of average glycemic indices. Glucose metabolism is influenced by numerous intrinsic rhythms and behaviors. There are likely many other elements in this complex multi-scale dynamic system of interactions as well, which highlight the importance of using analyses of variability in blood glucose levels across multiple temporal scales without the assumptions of linearity and stationarity. However, making casual inferences about the direction of influence between the elements in this complex system is difficult. Diabetes-related disruption of this complex system may manifests in multi-scale temporal glycemic variability and specific structural changes in the brain through complex dynamics not fully accounted for in the current investigation (one of many possibilities being mutli-scale temporal variability in circulating insulin levels).

There are several potential limitations of this study. This was a cross-sectional, observational study of a small yet well-characterized cohort. A larger validation study is therefore needed to determine prospectively whether reducing glycemic variability might improve outcomes and prevent cardiovascular adverse events associated with intensive glycemic levels lowering. As CGM sampling frequency was 5 minutes, we were also unable to study glycemic fluctuations with periods shorter than 30 minutes. In addition, the glycemic variability measures are derived from only 3 days of CGM data. To eliminate the influence of edge effect in EEMD technique and guarantee the accuracy of cycle length for each GVC, we only selected the GVCs with period less than 24 hours.

In summary, our results indicated that the relationships between glycemic variability and brain structure and function are time-scale dependent. The degree of glycemic variability within higher-frequency rhythms was associated with gray matter atrophy within the limbic system and temporo-parietal lobes (e.g. cingulum, insula, and hippocampus) and with worse cognitive performance. In diabetics, glycemic variability in lower frequency cycles were associated with worse learning and memory, more depression and longer DM duration. Implications are that diabetes management needs to target glycemic ultradian rhythms at specific frequencies in order to prevent brain atrophy and functional loss in older diabetic adults. Larger prospective studies are needed to determine whether time-scale dependent glycemic variability may serve as a new marker of diabetic complications, and in particular a marker of DM-related brain damage and subsequent loss of cognitive function.

## Supporting Information

Text S1 **EEMD-based decomposition for 72-hour continuous glucose monitoring (CGM) data.**
(DOCX)

Text S2 **The steps for calculation of the average period for each GVC.**
(DOCX)
