# Supplementary material for: Multi-Scale Glycemic Variability: A Link to Gray Matter Atrophy and Cognitive Decline in Type 2 Diabetes
Source: PLoS One. 2014 Jan 24;9(1):e86284. doi: 10.1371/journal.pone.0086284 (PMC3901681; doi:10.1371/journal.pone.0086284)
Supplement: Text S1 — EEMD-based decomposition for 72-hour continuous glucose monitoring (CGM) data. (DOCX) [file pone.0086284.s001.docx]

**Text S1.** EEMD-based decomposition for 72-hour continuous glucose monitoring (CGM) data

1. *Add white noise (amplitude equal to 5% of the standard deviation of raw CGM data) to each of the raw CGM data, to obtain a new data series.* This procedure solved the EMD problem of “mode mixing” (a single IMF may represent a combination of different time-scale or underlying physiological processes.), which occurs when oscillations with dramatically disparate scales appear within one IMF.

2. *Decompose the new data series into IMFs.* The envelopes of time series are derived via cubic spline by connecting local maxima and minima separately. Then, a sifting process decomposes the IMFs from the time series by subtracting the mean of envelopes. This process should repeat until the component satisfies two conditions:

(1) For the entire time series, the difference between numbers of local maxima and minima and zero-crossing must be either equal or differ at most by one (guarantees the IMFs are narrow band signals);

(2) At any data point, the mean value of upper and lower envelopes is zero.

The component that satisfies those two conditions is called an IMF. The difference between the time series and the k^th^ IMF is the k^th^ residue and will be treated as the data for decomposing the next IMF. Decomposition should be repeated until the residue becomes monotonic or only one maximum or minimum remains. The original data can be reconstructed by the summation of n IMFs and the n^th^ residue.

3. *Repeat steps 1 and 2 using different white noise series each time.*

4. *Calculate the (ensemble) means of corresponding IMFs of the decompositions as the final result.* The effect of the added white noise will always be able to be reduced to a negligibly small level by increasing the ensemble number. The ensemble number was 100 in this study.
