# Supplementary material for: Multi-Scale Glycemic Variability: A Link to Gray Matter Atrophy and Cognitive Decline in Type 2 Diabetes
Source: PLoS One. 2014 Jan 24;9(1):e86284. doi: 10.1371/journal.pone.0086284 (PMC3901681; doi:10.1371/journal.pone.0086284)
Supplement: Text S2 — The steps for calculation of the average period for each GVC. (DOCX) [file pone.0086284.s002.docx]

**Text S2.** The steps for calculation of the average period for each GVC

1. *First, we applied the Hilbert transform to the extracted GVCs (each GVC is denoted as S(t)) to calculate their instantaneous frequencies.* *S(t)* can be expressed as

$\text{S}(t)=A(t)\cos\varphi(t)$ (1)

The Hilbert transform of *S(t)* is defined as

$S_{H}(t)=\frac{1}{\pi}P\int\frac{S(t')}{t-t'}dt'$ (2)

where *P* denotes the Cauchy principal value.

2. $A$ *and $\varphi$ can be calculated by applying the Hilbert transform,*

$A(t)=\sqrt{S^{2}(t)+S_{H}^{2}(t)}$ (3)

$\varphi(t)=\arctan(S_{H}(t)/S(t))$ (4)

3. *Thus, the instantaneous frequency can be defined as*

$\omega=\frac{d\varphi(t)}{dt}$ (5)

4. *Therefore, the average cycle time is*

$T\text{=}\frac{2\pi}{\frac{1}{\text{a}}\int\omega dt}$ (6)

Here, $\text{a}$ is the total time period, and $dt$is the time interval of each input data. In this study, $\text{a}$= 72 hours and $dt$ is 5min.

5. *Finally, we calculated the average period of all subjects for each GVC. We totally got 6 GVCs for each subject and kept the first5 GVCs whose periods were less than 24 hours.*
